# Low or No Inhibitory Potency of the Canonical Galectin Carbohydrate-binding Site by Pectins and Galactomannans*

**DOI:** 10.1074/jbc.M116.721464

**Published:** 2016-04-26

**Authors:** John Stegmayr, Adriana Lepur, Barbro Kahl-Knutson, Matilde Aguilar-Moncayo, Anatole A. Klyosov, Robert A. Field, Stina Oredsson, Ulf J. Nilsson, Hakon Leffler

**Affiliations:** From the ‡ Section MIG (Microbiology, Immunology, Glycobiology), Department of Laboratory Medicine, Lund University, 221 00 Lund, Sweden,; the § Department of Biology and; ** Centre for Analysis and Synthesis, Department of Chemistry, Lund University, Lund, Sweden,; the ¶ Department of Biological Chemistry, John Innes Centre, Norwich Research Park, NR4 7UH Norwich, United Kingdom, and; ‖ Galectin Therapeutics, Newton, Massachusetts 02459

**Keywords:** cancer therapy, fluorescence anisotropy, galectin, glycobiology, polysaccharide, davanat, galactomannan, hemagglutination, pectasol, pectin

## Abstract

Some complex plant-derived polysaccharides, such as modified citrus pectins and galactomannans, have been shown to have promising anti-inflammatory and anti-cancer effects. Most reports propose or claim that these effects are due to interaction of the polysaccharides with galectins because the polysaccharides contain galactose-containing side chains that might bind this class of lectin. However, their direct binding to and/or inhibition of the evolutionarily conserved galactoside-binding site of galectins has not been demonstrated. Using a well established fluorescence anisotropy assay, we tested the direct interaction of several such polysaccharides with physiological concentrations of a panel of galectins. The bioactive pectic samples tested were very poor inhibitors of the canonical galactoside-binding site for the tested galectins, with IC_50_ values >10 mg/ml for a few or in most cases no inhibitory activity at all. The galactomannan Davanat® was more active, albeit not a strong inhibitor (IC_50_ values ranging from 3 to 20 mg/ml depending on the galectin). Pure synthetic oligosaccharide fragments found in the side chains and backbone of pectins and galactomannans were additionally tested. The most commonly found galactan configuration in pectins had no inhibition of the galectins tested. Galactosylated tri- and pentamannosides, representing the structure of Davanat®, had an inhibitory effect of galectins comparable with that of free galactose. Further evaluation using cell-based assays, indirectly linked to galectin-3 inhibition, showed no inhibition of galectin-3 by the polysaccharides. These data suggest that the physiological effects of these plant polysaccharides are not due to inhibition of the canonical galectin carbohydrate-binding site.

## Introduction

A range of plant polysaccharides is being investigated regarding their health-promoting effects; among them are pectin products like modified citrus pectin (MCP),^4^ *e.g.* GCS-100 and PectaSol-C®, and fractionated pectin powder (FPP) (1–24). One of the more exciting MCP effects reported was the prevention of cancer metastasis, reviewed by Glinsky and Raz (10). In addition, one study showed FPP to be superior over PectaSol-C® in anti-prostate cancer activity (8). Pectins, complex polysaccharides that are present in plant cell walls, are composed of a backbone containing 1,4-linked α-d-galacturonic acid (GalA), but they can be further organized into different classes with respect to the exact composition and substitution of the polysaccharide backbone (25). The two main types of pectins are the galacturonans and rhamnogalacturonan (RG)-I; the galacturans have a backbone of linear 1,4-linked α-d-GalA residues and can be unsubstituted (homogalacturonans) or substituted to various degrees with saccharides such as 2-*O*-methylxylose, 2-*O*-methylfucose, aceric acid, and several more in RG-II (not structurally related to RG-I), d-apiose in apiogalacturonan, and d-xylose in xylogalacturonan (25, 26). RG-I, which differs from the galacturonans in having a backbone built up by repeating units of [-α-d-GalA-1,2-α-l-Rha-1,4-]*_n_*, is more or less substituted at C-4 of the rhamnose (Rha) residues, depending on the species, with chains composed of either β-d-galactose (Gal) (galactans), α-l-arabinose (arabinans), or a mixture of both (arabinogalactans). The different classes of pectins should, however, not be considered as being separate molecules; on the contrary, they can be covalently attached to each other in many cases. It should also be pointed out that the exact composition of a pectin can vary considerably between different plant species, between plant tissue, and during development, making the pectins a highly complex family of polysaccharides (25–27). Because of this complexity and the high molecular mass of pectic polysaccharides, they are often modified by alkaline, temperature, acidic, and/or enzymatic treatments to produce a mixture of modified fragments with lower molecular mass (*e.g.* MCP, PectaSol-C®, and FPP) or to enrich for certain pectic domains, side chains, or monosaccharides (*e.g.* RG-I fragments or galactans) (1, 17, 21, 28–30). Another plant polysaccharide product, the galactomanann Davanat®, has also been proposed as a promising anti-metastatic drug. This large polysaccharide, with an average molecular mass of 60 kDa, is composed of a backbone of 1,4-linked β-d-mannose (Man) substituted with mono α-d-Gal via 1,6-linkage to the backbone (on average the ratio of Man/Gal is around 1.7) (31–34).

Regarding their mechanism of health promotion, these plant polysaccharides have been proposed to inhibit binding activities of galectins (in particular galectins-1 and -3) (2, 6, 10, 14, 18, 32) principally because galectins have a defining carbohydrate recognition domain (CRD) with established affinity for β-d-Gal residues (35–37) as found, to various degrees, in the plant polysaccharides mentioned above (*i.e.* in galactan side chains attached to pectic RG-I domains or Gal attached to the Man backbone of Davanat®). Moreover, galectins are involved in a number of cellular functions, such as regulation of intracellular glycoprotein trafficking, cell adhesion, cell signaling, and apoptosis with consequent effects in cancer, inflammation, and immunity (35–37), making them a reasonable functional target for the plant polysaccharides mentioned above. However, the actual interaction between galectins and most biologically active pectins or galactomannans has been analyzed biochemically to only a limited extent. Most proposals of galectin inhibition by pectin products are based on measurements in cell culture, where indirect effects on galectins cannot be ruled out (1–6, 9–16, 18, 19). In some studies, inhibition of hemagglutination was employed (17, 21, 38) but where other effects on the erythrocytes cannot be ruled out. In biochemical studies, one interacting partner was immobilized on a surface (2, 17, 21, 29, 38) where possible multivalent effects make estimation of specificity, affinity, or occupancy unclear because of the large size and heterogeneity of pectic saccharides. More detailed analysis of Davanat® by nuclear magnetic resonance (NMR) spectroscopy by Miller *et al.* (34, 39, 40) suggested interaction with galectin-1 and -3 at a site different from the canonical carbohydrate recognition site.

To test the interaction of these compounds with physiological concentrations of galectins in solution, we have now employed a fluorescent anisotropy (FA) assay that permits evaluation of their occupancy and affinity for the canonical galectin carbohydrate-binding site. Some of the compounds are further tested in cell-based assays for indirect measurement of galectin-3 inhibition in extra- and intracellular environments. Both the biochemical analysis using the FA assay and the cell-based assays show that, in contrast to expectations based on previous publications in the field, pectins and galactomannans are in fact poor inhibitors of galectin/carbohydrate interactions and thereby contradicting the notion found in the literature stating that pectins and galactomannans are potent and selective inhibitors of the galectin CRD.

## Experimental Procedures

### 

#### 

##### Materials

Chemicals including fluorescent probes and galectins were as described before (41–44), unless stated otherwise. FPP was from Thorne Research (Dover, ID); PectaSol-C® was from Eco Nugenics (Santa Rosa, CA), and l-arabinose and Davanat® was from Sigma, Lot 042K7277 (custom-made product D7065). One preparation of MCP was kindly provided by MacKinnon *et al.* (20) (University of Edinburgh, Edinburgh, Scotland, UK). RG-I (potato), pectic galactan (potato), galactobiose (β-1,4 and β-1,3 ratio ∼2:1), 6^1^-α-d-galactosyl-mannotriose, and 6^3^,6^4^-α-d-galactosyl-mannopentaose were received from Megazyme International Ireland (Wicklow, Ireland). Another preparation of MCP as well as RG-I (ginseng), RG-I-4 (ginseng), and pectic galactan preparations from potato named p-oligo were as described in Gao *et al.* (21). Details of the chemical synthesis of galacto-oligosaccharides (Galβ1–3Gal; Galβ1–4Gal; Galβ1–6Gal; Galβ1–3Galβ1–6Gal; Galβ1–6Galβ1–6Gal; branched Galβ1–6(Galβ1–3)Gal, and Galβ1–6(Galβ1–3)Galβ1–6Gal), will be reported elsewhere^5^ (John Innes Centre, Norwich Research Park, Norwich, UK). 3,3′-Dideoxy-3,3′-bis-[4-(3-fluorophenyl)-1*H*-1,2,3-triazol-1-yl]-1,1′-sulfanediyl-di-β-d-galactopyranoside (33DFTG) was kindly provided by Galecto Biotech AB (Copenhagen, Denmark). NDP52 conjugated to fluorescein, FITC-aminohexanoic acid-PGLAYGNPYSGIQE, was purchased from Peptron Inc. (Daejeon, South Korea). Hen blood in Alsever's solution was purchased from Håtunalab AB (Bro, Sweden). ID-Cards “NaCl, enzyme test, and cold agglutinins” (gel cards produced by DiaMed GmbH (Cressier FR, Switzerland)) and ID-CellStab solution were received from Svenska Labex AB (Helsingborg, Sweden), and 2.5% trypsin in phosphate-buffered saline (PBS) was bought from VWR International AB (Stockholm, Sweden). Glycyl-l-phenylalanine 2-naphthylamide (GPN) was purchased from Santa Cruz Biotechnology, Inc. (Dallas, TX). The human breast carcinoma cell line JIMT-1 was purchased from American Type Culture Collection (ATCC, LGC standards AB, Borås, Sweden). DMEM/Ham's F-12 medium (Biochrom GmbH), fetal bovine serum (FBS) (Biochrom GmbH), penicillin/streptomycin solution (Biochrom GmbH), nonessential amino acids (Biochrom GmbH), and insulin were bought from VWR International AB.

##### Fluorescence Anisotropy Assay

FA was used to analyze interactions of probes and ligands with galectins in solution, as described before (41–44). A PheraStarFS plate reader, with software PHERAstar Mars version 2.10 R3 (BMG, Offenburg, Germany), was used, and FA of fluorescein-tagged saccharide probes was measured with excitation at 485 nm and emission at 520 nm.

The anisotropy (A) is given in units of mA, where the theoretical maximum is 400, and the value for free fluorescein typically 23. All conditions were measured in at least two duplicate wells. This showed that the anisotropy measurement is highly reproducible, with between well difference of >5 mA occurring less than 10% of the time and >10 mA less than 1% of the time.

**FIGURE 1.**
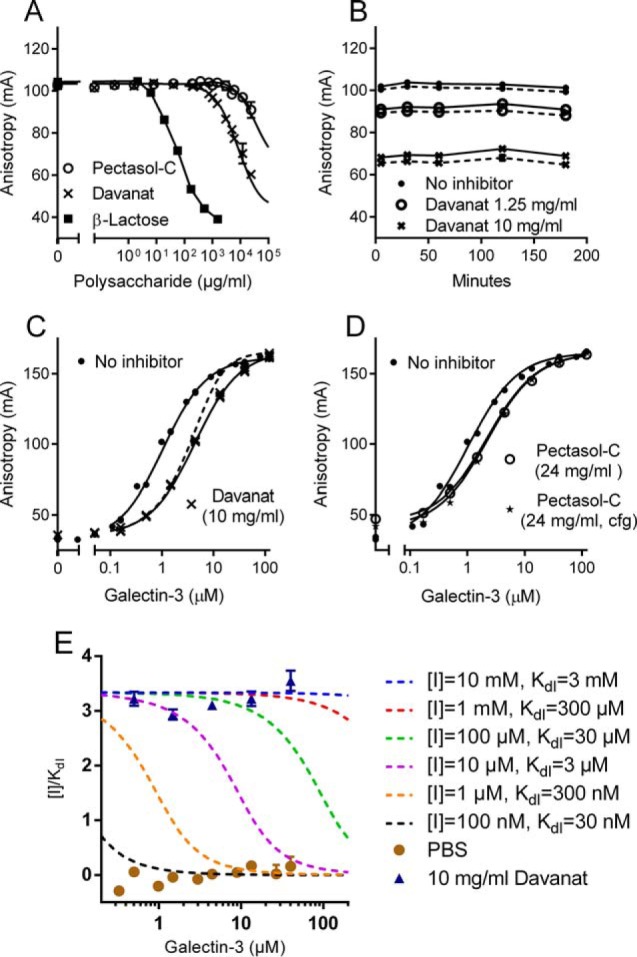
**Analysis of galectin-3 interaction with Davanat® and PectaSol-C®.** *A,* inhibition of galectin-3 (1 μm) binding to A-tetra-probe (0.1 μm) by increasing concentrations of Davanat®, PectaSol-C®, or β-lactose (as reference) measured by the FA assay 5 min after mixing components. The best fit calculated curves correspond to IC_50_ values of 8 mg/ml for Davanat®, 50 mg/ml for PectaSol-C®, and 0.07 mg/ml for β-lactose. *B,* time course of galectin-3-Davanat® interaction. The experiment was done as for *A*, but the results are shown for three Davanat® concentrations (0, 1.25, and 10 mg/ml) as read after different incubation times (5, 30, 60, 120, and 180 min) as indicated. The experiment was done in the presence (*dashed line*) or absence (*unbroken line*) of β-mercaptoethanol. *C,* binding of increasing concentrations of galectin-3 (*x* axis) to fluorescent A-tetra probe (0.1 μm) without (*filled circles*) or with Davanat® (10 mg/ml, ∼200 μm) as inhibitor (× *symbols*). Theoretically calculated binding curves are shown for no inhibitor (*solid line*), 10 mm inhibitor of *K_d_* 3 mm (*2nd solid line*) or 10 μm inhibitor of *K_d_* 3 μm (*broken line* that does not fit data points). Both concentration-*K_d_* combinations agree with the inhibitory potency calculated from *A. D,* binding of increasing concentrations of galectin-3 (*x* axis) to fluorescent A-tetra probe (0.1 μm) without (*filled circles*) or with centrifuged PectaSol-C® (24 mg/ml) (*) or non-centrifuged PectaSol-C® (*open circles*). Theoretically calculated binding curves are shown for no inhibitor (*solid line*) or 24 mm inhibitor of *K_d_* 20 mm (*2nd solid line*), in agreement with the inhibitory potency calculated from *A*. PectaSol-C® at 24 mg/ml contained a precipitate, but there was no difference when it was removed by centrifugation. *E*, [I]/*K_d_*_I_ = [IG]/[G] plotted against the added galectin concentration, where [G] and [I] are the concentrations of free galectin-binding sites on inhibitor, respectively, and [IG] is the concentration of galectin bound to inhibitor, and *K_d_*_I_ is the dissociation constant for the galectin-inhibitor interaction at each site. *Triangle symbols* represent the [I]/*K_d_*_I_ values for 10 mg/ml Davanat®, and the *circle symbols* represent the values for no inhibitor (*i.e.* PBS) and are calculated from the values found in *C*. Model curves (*dashed lines*) for different pairs of [I] and *K_d_*_I_, all with an ratio of 3.3, are also included for comparison. The model curves bend down toward a ratio of zero when the galectin-binding sites on the inhibitor start to become saturated. Hence, the lower the concentration of sites, the more to the left the downturn of the curve will be. All experiments were done at least in duplicate, and *bars* showing the standard error are included but are not visible in most cases as they are smaller than the symbols. *A* and *C* in addition show the combination of two different dilution series of inhibitor analyzed at separate occasions.

In the presence of increasing amounts of galectin, the FA increases from the value for unbound probe (A_0_ typically about 35 mA) to the value when all probe is bound by galectin (A_max_, typically between 150 and 250 mA, depending on which galectin was analyzed) (see *left curve* in Fig. 1*C*). In this study, the effect of inhibitors of the saccharide probe/galectin interaction was tested in two ways. In the first, a fixed low concentration of fluorescein-tagged probe (0.1 μm) and galectin were mixed with a range of concentrations of inhibitor. An optimal probe was chosen for each galectin to permit use of as low as possible concentration of galectin, while still achieving a sufficient rise in anisotropy for a reliable measuring range (42–44). Typically, the concentration chosen gave about half of the probe bound in absence of inhibitor, making % inhibition correlating roughly linearly with galectin-binding sites (Fig. 1*A*). With structurally defined inhibitors (pure synthetic galacto-oligosaccharides, small galactomannans, and reference ligands) the molecular mass, molar concentrations, and number of binding sites (in this case one binding site per ligand) were known and their dissociation constants (*K_d_*) could therefore be directly calculated, using the data from the FA assay as described in Sörme *et al.* (41). With polysaccharide inhibitors where the molecular mass and number of binding sites were not known, the inhibitor concentration (IC_50_ in mg/ml) giving 50% reduction of the measured end point (anisotropy) was calculated, where 0% inhibition is anisotropy in the absence of inhibitor, and 100% inhibition is estimated as the anisotropy of the free probe (that is as expected when all galectin is bound by inhibitor). Even if the molar concentration of the inhibitor is not known, the anisotropy value at a given condition permits calculation of the molar concentration of free galectin, as described below, and from this what fraction of the galectin is bound by a given mass per volume concentration of inhibitor. From this, the ratio of the molar concentration of the free inhibitor binding sites to their *K_d_* value can be calculated, even if the FA assay cannot distinguish between the case when this is due to a few high affinity binding sites in the polysaccharide fraction or many of low affinity.

In the next assay, a fixed low concentration of fluorescent probe (0.1 μm) was mixed with a fixed concentration of inhibitor and a range of concentrations of galectin (Fig. 1*C*). Without inhibitor, this gives a curve going from anisotropy for free fluorescent probe (A_0_) to the value for all probes bound by galectin (A_max_) as described above. In the presence of inhibitor, the curve shifts to the right and may or may not change slope, and it was compared with model binding curves as described under “Results.” The data were also used to estimate concentration and affinity of binding sites on saccharide inhibitor using the following derivations.

From the law of mass action for galectin interaction with the fluorescent probe (P), it follows that the concentration of free galectin [G] = K*_d_*_P_ ([GP]/[P]), where [GP] is the concentration of fluorescent probe bound by galectin. This can be calculated from the measured anisotropy because [GP]/[P] = (A − A_0_)/(A_max_ − A), and galectin affinity for the probe, *K_d_*_P_, is determined by analysis in the absence of inhibitor as described by Sörme *et al.* (41). The value of [G] is used to calculate the ratio of free binding sites on the inhibitor (I) with their affinity, [I]/*K_d_*_I_ = [IG]/[G] = ([G_tot_]/[G]) − 1 − ([GP]/[G]), because [IG] = [G_tot_] − [G] − [GP]; the last term can also be calculated from the measured anisotropy value and the equations given above, even if it is insignificantly small compared with the other terms when the concentration of probe is small compared with the galectin concentrations used. The ratio [I]/*K_d_*_I_ derived in this way was plotted (*y* axis) against total added galectin (*x* axis) as shown in Fig. 1*E*, in an attempt to estimate the average affinity of the binding sites found in Davanat® and PectaSol-C®.

A third variant of the FA assay was used to investigate the interaction of inhibitors with a second non-carbohydrate-specific binding site identified for galectin-8 (45, 46). This second site, located on the convex side of the C-terminal CRD of galectin-8, has strong affinity for a peptide from the protein NDP52 (*K_d_* in the low micromolar range). By using this peptide conjugated to fluorescein as probe in the FA assay (46) instead of using fluorescent saccharide probes, as described above, we could study the interaction of inhibitors with this non-canonical binding site. These experiments were carried out in triplicate, and a concentration of 0.1 μm fluorescein-conjugated NDP52 peptide was used in all experiments. To establish A_max_ values, galectin-8 was titered up to a 20 μm concentration. A concentration of 0.2 μm galectin-8 was used in the inhibition experiments, where the inhibitors were tested in concentrations ranging from 30 to 1670 μg/ml.

##### Galectin-3-induced Hemagglutination Assay

Hemagglutination by galectin-3 was investigated in two assays, hemagglutination in V-shaped microplates (96 wells) and hemagglutination in gel cards described by Nowak *et al.* (47) and Lapierre *et al.* (48), respectively. Briefly, fresh hen blood in Alsever's solution was washed with 0.15 m NaCl, whereupon a 4% erythrocyte solution (by volume in PBS) was prepared and incubated with 1 mg/ml trypsin at 37 °C for 1 h. The cells were then washed with 0.15 m NaCl. The erythrocytes were diluted and preserved as a 10% solution in ID-CellStab solution. For the hemagglutination in V-shaped microplates, 1 volume of erythrocyte suspension in PBS (final concentration 0.6%), 1 volume of galectin solution, 1 volume of 0.15 m NaCl with or without the galectin inhibitor, and 1 volume of 1% bovine serum albumin (BSA) in 0.15 m NaCl were added to each well of the microplate. The plate was then shaken vigorously for 90 min at room temperature and then left standing still for at least 90 min before observing the hemagglutination status in each well. For the hemagglutination in gel cards, 1 volume of the inhibitor dissolved in 1% BSA in 0.15 m NaCl, 1 volume of galectin dissolved in 0.15 m NaCl, and 1 volume of erythrocyte suspension (final concentration 0.53%) in 0.15 m NaCl were mixed and shaken for a short period in U-shaped microplates at room temperature. Each mixture was then transferred to individual gel card wells, whereupon the cards were centrifuged for 10 min at 85 × *g* in an ID-Centrifuge (DiaMed GmbH) and thereafter photographed and analyzed for hemagglutination.

##### Calculations of the Relationship between MIC Value, Galectin-3 Concentration, and Inhibitor and Receptor Affinities

We assume that the effect (*e.g.* hemagglutination) of a galectin, G, is governed by its binding to a receptor, R, to form the complex RG. We asked how much galectin inhibitor, I, competing with this receptor will be needed to prevent the effect. In a first case we assume, for simplicity, that >50% saturation of the receptor is required to see an effect. Hence, we asked what concentration of added inhibitor, [I_tot_], is required to bring down receptor saturation to this threshold of 50% that is minimal inhibitory concentration (MIC) of the effect, under different conditions of added galectin concentration [G_tot_], available receptor concentration [R_tot_], and affinities of the galectin/receptor interaction (*K_d_*_R_) and galectin/inhibitor interaction (*K_d_*_I_). Please note that MIC defined this way is not the same as IC_50_ mentioned above, which instead is the inhibitor concentration reducing initial galectin-induced anisotropy (probe binding) by half, which is of some value less than 100%.

The interactions will be governed by Equations 1 and 2 of mass action,

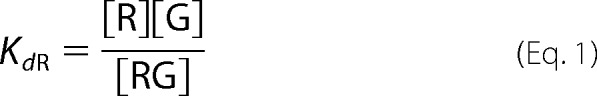


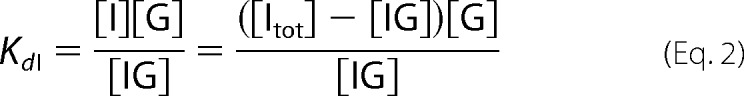
 at 50% receptor saturation [R] = [RG] and Equation 1 can be rewritten as Equations 3 and 4,

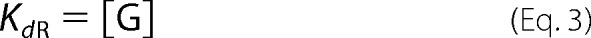
 and

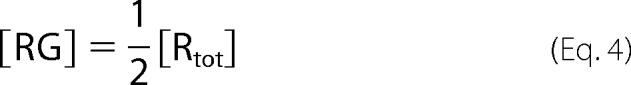
 and by inserting Equation 3 into Equation 2 and rearranging gives Equation 5

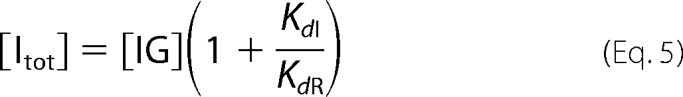
 and from [G_tot_] = [G] + [IG] + [RG] and Equations 3 and 4 follows Equation 6,

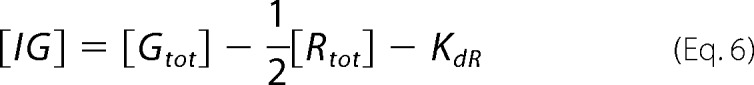
 which after insertion to Equation 5 gives Equation 7

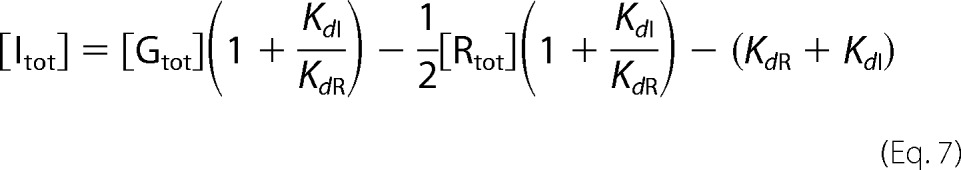


Thus plotting [I_tot_] = MIC (*y* axis) as a function of concentration of added galectin [G_tot_] (*x* axis) will give a straight line with a slope of 1 + (*K_d_*_I_/*K_d_*_R_). The weaker the inhibitor, *i.e.* higher *K_d_*_I_, the steeper the line will be. The line will cross the *x* axis at the point where just the added galectin by itself gives 50% receptor saturation; hence, [I_tot_] = 0 and [G_tot_] = 1/2[R_tot_] + *K_d_*_R_ (from Equation 6). An interesting consequence is that if the galectin concentration is just a small amount above this level, by increasing the receptor saturation a little above 50% (barely enough to see the measured effect) the concentration of inhibitor required to bring it down to 50% or below may be very small even if the inhibitor has low affinity for the galectin (*i.e.* high *K_d_*_I_).

In a more general analysis, one can consider cases when the receptor saturation required for an effect is a number other than 50%, *i.e.* [R]/[RG] = *Q*, where *Q* = 1 only in the special case of 50% receptor saturation. Then Equation 3 becomes *K_d_*_R_/*Q* = [G], and Equation 4 becomes [RG] = [R_tot_]/(1 + *Q*), which after insertion into Equations 5–7 gives Equation 8,

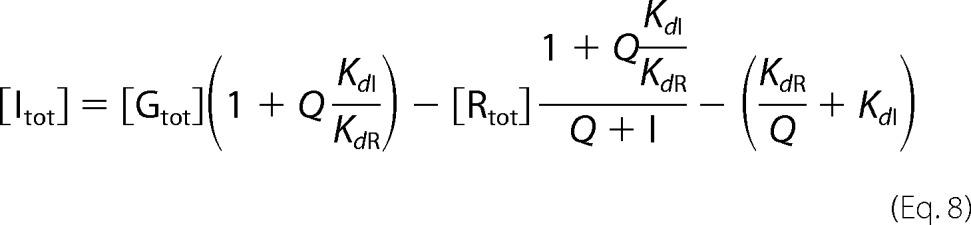


This has two interesting consequences. First, the slope of the line [I_tot_] *versus* [G_tot_] will be steeper for cases where lower receptor saturation is required for effect, with *Q* > 1. Second, if two relatively weak inhibitors are compared, the relative slopes of the two lines will be independent of *Q*, when the *Q*(*K_d_*_I_/*K_d_*_R_) term in the slope is large enough so the 1 can be ignored. At the border of effect, when [I_tot_] = 0, now [G_tot_] = [R_tot_]/(1 + *Q*) + (*K_d_*_R_/*Q*). Thus a higher *Q* will give an intercept of the *x* axis for at lower value of [G_tot_].

##### Cell Culture

JIMT-1 breast cancer cells were maintained in Dulbecco's modified Eagle's medium (DMEM)/Ham's F-12 medium mixture (1:1) supplemented with 10% FBS, 100 units/ml penicillin, 100 μg/ml streptomycin, 1 mm nonessential amino acids, and 10 μg/ml insulin. The cells were kept at 37 °C in a humidified incubator supplemented with 5% CO_2_ in air and were sub-cultured twice every week.

##### Inhibition of GPN-induced Galectin-3 Accumulation

JIMT-1 cells were seeded in multi-well plates, with sterile glass coverslips in each well, at a cell density of 29,000 or 43,000 cells/cm^2^ and allowed to attach to the surface for 48 or 24 h, respectively, before treatment with Davanat®, FPP, or 33DFTG. Davanat® and FPP were dissolved in DMEM/Ham's F-12 medium without supplements at a concentration of 5.75 mg/ml, and the solution was then sterile-filtered, and the supplements (10% FBS, 100 units/ml penicillin, 100 μg/ml streptomycin, 1 mmol/liter nonessential amino acids, and 10 μg/ml insulin) were thereafter added resulting in a final concentration of Davanat® and FPP of 5 mg/ml. Further dilutions of Davanat® or FPP to 0.5 mg/ml were done using supplemented DMEM/Ham's F-12 medium. 33DFTG was dissolved in 99.7% cell culture grade dimethyl sulfoxide (DMSO) and diluted further in supplemented DMEM/Ham's F-12 medium to final concentrations of 1 or 10 μm with concentrations of 0.1% DMSO in both cases. The JIMT-1 cells were treated with either 0.5 or 5 mg/ml Davanat® or FPP or with 1 or 10 μm 33DFTG for 24 h, whereupon the cells were treated with 0.3 mm GPN for 12 min. Cells treated with supplemented DMEM/Ham's F-12 medium were used as control for Davanat®- and FPP-treated cells, whereas supplemented DMEM/Ham's F-12 medium with 0.1% DMSO was used for 33DFTG. The cells were, after the GPN treatment, quickly washed with PBS and fixed with 3.7% formaldehyde for 15 min before blocking/permeabilization with 1% Tween® 20 and 1–5% BSA in PBS for at least 45 min. The cells were then stained with a primary rat anti-mouse galectin-3 antibody (49, 50) for 1 h before washing with PBS and staining with a secondary goat anti-rat IgG Alexa Fluor® 594-conjugated antibody for an additional hour. The cell nuclei were visualized by staining with the DNA-binding fluorescent agent bisbenzimide (Hoechst). The coverslips were finally mounted on glass slides using Mowiol® 4-88 and left at 8 °C overnight to solidify before examination. The samples were imaged with an LSM-510 confocal laser scanning microscope (Carl Zeiss Microscopy GmbH, Oberkochen, Germany) equipped with a Hamamatsu R6357 photomultiplier (Hamamatsu Photonics K.K., Hamamatsu, Japan). The Alexa Fluor® 594-conjugated antibody was excited using a 561-nm diode-pumped solid-state laser, and a 575-nm long pass filter was used for the emission. For the excitation of the bisbenzimide, a 405-nm diode-pumped solid-state laser was used, and a bandpass filter with the range of 420–480 nm was used for the emission. Z-stacks composed of single optical planes in high magnification were collected and used to visualize the cells using the software ZEN 2012 (black edition), version 8.0 (Carl Zeiss Microscopy GmbH). Maximum intensity projections of each picture were analyzed with ImageJ version 1.47 (W. S. Rasband, ImageJ, National Institutes of Health, Bethesda) to quantify the number of galectin-3 dots/nuclei in each picture.

##### Turbidimetry Assay

Turbidimetry was measured as absorption at 350 nm in the PheraStarFS plate reader and was used to quantify formation of galectin-ligand precipitates, with calculations of bound and unbound concentrations and affinities as described before (51, 52).

## Results

### 

#### 

##### No or Low Binding of the Canonical Carbohydrate-binding Site of the Galectin by Pectins and Galactomannans

The FA assay directly measures the % binding of a fluorescein-tagged saccharide probe to a galectin, which binds in the galectin-defining canonical β-galactoside-binding sites, as shown by galectin mutants, x-ray crystallography, and inhibition by classical galectin ligands, and described in detail in Refs. 41–44, 51, 52. The inhibition of the binding of the probe to the galectin by a compound therefore measures the potency of this compound (inhibitor) to compete for or in another way prevent binding to the canonical galectin β-galactoside-binding site. Here, we first applied this assay to different pectin-derived fractions (PectaSol-C®, FPP, MCP, RG-I, RG-I fragment (RG-I-4), and galactans) and a galactomannan (Davanat®).

**FIGURE 2.**
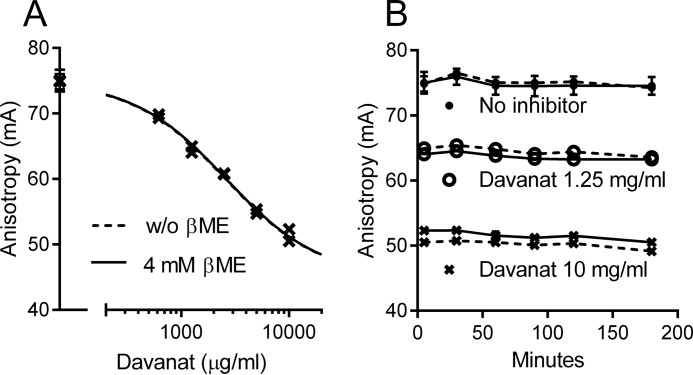
**Analysis of galectin-9N interaction with Davanat®.** *A,* inhibition of galectin-9N (1 μm) binding to the lacto-*N*-neotetraose probe (0.1 μm) by increasing concentrations of Davanat® in the presence or absence of β-mercaptoethanol (βME), measured by the FA assay 5 min after mixing components. The best fit calculated curves correspond to an IC_50_ value of ∼3 mg/ml. *B,* time course of galectin-3/Davanat® interaction. The experiment was done as for *A*, but the results are shown for three Davanat® concentrations (0, 1.25, and 10 mg/ml) as read after different incubation times (5, 30, 60, 120, and 180 min) as indicated. The experiment was done in the presence (*dashed line*) or absence (*unbroken line*) of βME. All experiments were done in at least duplicate, and *bars* showing standard error are included but are not visible in most cases as they are smaller than the symbols.

Fixed low concentrations of galectin and fluorescein-tagged probe were mixed with a range of concentrations of an inhibitor; fluorescence anisotropy was measured for each data point, and inhibition curves were calculated as exemplified for galectin-3 in Fig. 1*A* and galectin-9N in Fig. 2*A*. Even with these large polysaccharides, equilibrium was obtained after 5 min after mixing of components, as we found sufficient for previously analyzed inhibitors, because values did not change after extended incubation times up to 3 h (Figs. 1*B* and 2*B*).

The IC_50_ values were calculated for each putative inhibitor, and the data are compiled in Table 1. Because the polysaccharides are not precisely defined molecularly, but rather as mixtures of compounds with an average molecular mass, their concentration (*x* axis in Figs. 1*A* and 2*A*) and IC_50_ values are given in micrograms/ml. An inhibition curve for β-Lac (in μg/ml) is also shown for comparison (Fig. 1*A*).

**TABLE 1:** **Estimated IC_50_ values (mg/ml) for pectic samples, Davanat®, and β-lactose (Lac) as a reference ligand toward different galectins** About 1 μm of each galectin with 0.1 or 0.02 μm of appropriate fluorescence probe was tested in the presence of a range of concentrations of the different compounds; anisotropy was measured, and IC_50_ values were estimated. NI indicates no inhibition at the highest tested concentration. Blank field indicates not tested.

Samples	Highest concentration tested	Galectins								
		Gal-1	Gal-2	Gal-3	Gal-4C	Gal-4N	Gal-7	Gal-8N	Gal-9C	Gal-9N
	*mg/ml*									
**Pectins**										
PectaSol-C®	10 (24)	NI*^a^*	NI	>24	NI	NI	NI	>24	NI	NI
FPP	6.6	NI*^a^*		NI	NI	NI		>7	NI	NI
MCP (20)	10	NI*^a^*	NI	NI	NI	NI		>10	NI	NI
MCP (17)	2	NI*^a^*		NI						

**RG**										
RG-I (potato)	20	>20*^a^*	5–10	>20	>20	>20		5	>20	>20
RG-I (ginseng)	2	NI*^a^*		≫2						
RG-I-4	2	NI*^a^*		≫2						

**Pectic galactans (potato)**										
Pectic galactan	10	NI*^a^*	>10	NI	NI	NI		>10	NI	NI
p-oligo (7.3 to 65.3-mer, average 3.2 kDa)	1	≫1*^a^*		NI						
p-oligo-H (13.5-mer, 2.2 kDa)	1	NI*^a^*		≫1						
p-oligo-J (25.2-mer, 4.1 kDa)	1	NI*^a^*		NI						
p-oligo-K (34.5-mer, 5.6 kDa)	1	NI*^a^*		NI						
p-oligo-L (46.8-mer, 7.6 kDa)	1	≫1*^a^*		NI						

**Galactomannans**										
Davanat®	24	>20*^a^*	>20	10	10	>20	>20	10	10	3

**Reference ligands**										
β-Lac	1.5			0.07						

*^a^* Data were measured in the presence of 4 mm β-mercaptoethanol.

The data show that all the modified pectin mixtures are poor galectin inhibitors in this assay (Table 1). Pectasol-C® was tested up to 10 mg/ml and showed weak inhibitory potency for galectin-3 and the N-terminal CRD of galectin-8 (galectin-8N), but the compound inhibited no other galectin at this concentration. Pectasol-C® was also tested at up to 24 mg/ml with galectin-3 (Fig. 1*A*) and showed the expected small increase in inhibition. A precipitate formed at these higher concentrations, but this did not affect galectin binding, as the inhibition was the same after removal of the precipitate by centrifugation. FPP was only tested up to 6.6 mg/ml because a colored contaminant disturbed the analysis at higher concentrations. FPP inhibited only galectin-8N but no other galectin at the highest concentration tested. The MCP sample, as used in MacKinnon *et al.* (20), was tested at concentrations ranging from 0.31 to 10 mg/ml for galectin-3 and 1.25 to 10 mg/ml for galectin-1, -2, -4N, -4C, -8N, -9N, and -9C, but it showed no inhibition of the galectins tested, except a modest inhibition of galectin-8N (below the IC_50_ at the highest concentration tested). As the composition of different MCPs may differ depending on the source, extraction, and post-extraction treatments, for example, one additional MCP sample obtained from Gao *et al.* (17) was tested against galectin-1 and -3, but no inhibition was observed for the concentrations tested (0.78–2000 μg/ml). The only pectin domain containing β-Gal residues is RG-I (mainly 1,4-linked β-Gal residues), and this domain has therefore been postulated to be responsible for the galectin inhibition. The isolated RG-I domain, or fragments of it, was also suggested to inhibit galectin-3 in the low microgram/ml range (21, 27, 30). Two different RG-I samples of different origin (potato and ginseng) and one sample of an RG-I fragment (RG-I-4, reported to have high affinity for galectin-3 (21)) were tested for galectin inhibition. The potato RG-I sample was tested at concentrations ranging from 1.25 to 20 mg/ml, but only modest inhibition of galectin-2 and -8N could be observed with predicted IC_50_ values around 5 mg/ml. Ginseng RG-I and RG-I-4 were tested against galectin-1 and -3 at concentrations ranging from 500 to 2000 μg/ml and 0.32–2000 μg/ml, respectively. The RG-I and RG-I-4 only showed modest inhibition of galectin-3 at the highest concentration, similar to the potato RG-I at the same concentration, and no inhibition of galectin-1 at the highest concentration (2 mg/ml). The next set of samples tested were pectic galactans, *i.e.* one of the side chain found attached to the RG-I domain. One galactan tested was commercial pectic galactan from potato (prepared through alkaline extraction and enzymatically treated to remove other polysaccharides), shown to contain mainly 1,4-linked β-d-Gal residues (29). Another set of pectic galactans tested were p-oligo and p-oligo H-L described in Gao *et al.* (21). Briefly, pectic galactan from potato was subjected to partial acid hydrolysis to produce long-chain galactans, whereupon the neutral fraction (p-oligo) was obtained by anion exchange chromatography. Sub-fractions of p-oligo (H-L) were prepared to have more defined molecular mass distributions. The results show that the pectic galactan samples had no or very weak inhibitory effects of galectin-1 or -3 at the concentrations tested (0.6–10 mg/ml for the commercial pectic galactan and 0.25–1 mg/ml for the p-oligo samples).

The galactomannan Davanat® had a better binding profile compared with the pectic samples, and it inhibited all galectins with IC_50_ values in the 10 mg/ml range or higher, except for slightly better binding for the N-terminal CRD of galectin-9 (galectin-9N) (Fig. 2*A*). All the polysaccharides had much lower inhibitory potency (on a mass per volume basis) than β-Lac (IC_50_ value of 70 μg/ml for galectin-3) (Fig. 1*A* and Table 1).

##### Estimation of Number and Affinity of Galectin-binding Sites in Polysaccharide Fractions

As explained under “Experimental Procedures,” the FA assay described above cannot distinguish between the case when inhibitory potency is due to few high affinity binding sites in the polysaccharide fraction, or many of low affinity. To look for high affinity binding sites, we instead titered increasing concentrations of galectin into a fixed high concentration of polysaccharide, with a small concentration of fluorescent probe as a tracer to determine concentration of free galectin (Fig. 1, *C–E*). In the absence of inhibitor, the anisotropy values follow a curve (sigmoidal in the semi-log plot as shown in Fig. 1*C*) going from the value for the free probe (35 mA in Fig. 1, *C* and *D*) to a value when the probe is completely bound to the galectin (about 165 mA). With Davanat® (Fig. 1*C*) and PectaSol-C® (Fig. 1*D*), the curves shifted to the right, reflecting binding of the galectin to the inhibitor. To see this more clearly, [I]/*K_d_*_I_ was calculated from the anisotropy values, as described under “Experimental Procedures,” and plotted against the added galectin concentration in Fig. 1*E*. As expected, the values in the absence of inhibitor were ∼0. With 10 mg/ml Davanat®, the values were about 3.3 for all galectin concentrations used in the analysis. This shows that there was no significant saturation of any galectin-binding site on the inhibitor even at the highest galectin concentration. Had there been saturation, the curve would have turned down and approached the zero level, as illustrate by the model curves (*dashed lines*) in Fig. 1*E*. The ratio of about 3 for [I]/*K_d_*_I_ also agreed with the inhibitory potency of Davanat® in Fig. 1*A* and the size of the shift between the curves in Fig. 1*C*, and no evidence for higher affinity sites was evident in the latter as illustrated by the steeper dotted model curve that does not fit the data. Thus, 10 mg/ml Davanat® contains at least 1 mm galectin-binding sites with *K_d_* values of at least 300 μm, but we cannot distinguish this from cases with even higher concentrations of binding sites with correspondingly lower affinity (higher *K_d_*). With the proposed molecular mass of Davanat® of 40–60 kDa (33), 10 mg/ml of the compound would correspond to ∼200 μm, with at least five galectin-binding sites per molecule. In a similar way, the data with 24 mg/ml PectaSol-C® (Fig. 1*D*) gave an [I]/*K_d_*_I_ of about 1.2, which shows much weaker binding of PectaSol-C® compared with Davanat®, and no sign of saturation at the highest galectin concentration tested. With the proposed molecular mass of PectaSol-C® of 10–20 kDa, 24 mg/ml of the compound would correspond to around 1.2–2.4 mm with, for example, 5–10 binding sites per molecule with average *K_d_* values of 10–20 mm. The other polysaccharides did not have enough inhibitory potency, at the highest concentrations available, to make this type of analysis possible. They all had no or < 20% inhibition, as a safe reliably detectable minimum inhibition level, in the assay type exemplified in Fig. 1*A* and 2*A*. From this one can calculate that they had an [I]/*K_d_*_I_ ratio of <1 at the highest concentration tested.

##### Analysis of Galectin Inhibition by Pectins/Galactomannans, in Light of Possible Structural Components

Next, we analyzed the galectin affinity (*K_d_*) of known structural components of the plant polysaccharides, made synthetically or obtained as defined fragments, and also included some reference compounds (Table 2).

**TABLE 2:** **Calculated *K_d_* values (μm) for synthetic galacto oligo-saccharides, galactomannans, and reference ligands towards different galectins** About 1 μm of each galectin with 0.1 or 0.02 μm of appropriate fluorescence probe was tested in the presence of a range of concentrations of the different compounds; anisotropy was measured, and *K_d_* values were calculated. NI indicates no inhibition at highest concentration tested. Blank field indicates not tested. NA means not applicable.

Samples	Highest concentration tested	Galectins								
		Gal-1	Gal-2	Gal-3	Gal-3 R186S	Gal-4C	Gal-4N	Gal-8N	Gal-9C	Gal-9N
	μ*m*									
**Synthetic galacto-oligosaccharides**										
Galactobiose (β-1,4 and β-1,3 ratio ∼2:1)	10,000	1100*^a^*	2100	280	990	2700	1600	270	290	91
Galβ1–3Gal	2000	3500*^a^*		180						150
Galβ1–4Gal	2000	NI*^a^*		NI						1200
Galβ1–6Gal	2000	NI*^a^*		3100						NI
Galβ1–3Galβ1–6Gal	2000	1200*^a^*		110						68
Galβ1–6Galβ1–6Gal	2000	NI*^a^*		NI						NI
Branched Galβ1–6(Galβ1–3)Gal	2000	1300*^a^*		130						100
Branched Galβ1–6(Galβ1–3)Galβ1–6Gal	2000	800*^a^*		80						51

**Galactomannans**										
6^1^-α-Galactosyl-mannotriose	10,000		NI	3600		NI	NI	7400		2400
6^3^,6^4^-α-Galactosyl-mannopentaose	10,000	4300*^a^*	8300	1500		NI	12 000	2500	2000	980

**Reference ligands**										
Methyl β-Gal	40,000	5500*^a^*		3000		26,000		3700	8600	4000
Methyl β-Lac	4000	170*^a^*		110	290	2900	2600		190	
Methyl β-LacNAc	1000	84*^a^*		52	1200					
β-Lac	4500			93						
l-Arabinose	2 × 10^5^	NI*^a^*	1.9 × 10^5^	1.7 × 10^5^		NI	2.6 × 10^5^	1.1 × 10^5^	1.5 × 10^5^	0.94 × 10^5^
33DFTG	NA	0.01*^b^*		0.014^§^						

*^a^* Data were measured in the presence of 4 mm β-mercaptoethanol.

*^b^* Data are from MacKinnon *et al.* (55).

The galactomannan Davanat® has a Man backbone with Gal monosaccharide side chains. Galactosylated tri- and penta-mannosides representing this structure inhibited galectins with about the same potency as free Gal, with *K_d_* values in the millimolar range. With a *K_d_* value of about 3 mm per site, the calculation based on Fig. 1*E*, described above, would give about 10 mm sites per 10 mg/ml Davanat® or about 50 sites per 50-kDa molecule, which agrees well with published chemical data (34, 39).

Finally, we tested synthetic di- to tetra-galactans, proposed as components of pectic galactans, as inhibitors of galectins-1, -3, and -9. The major component found in pectic galactans, Galβ1–4Gal, had no inhibitory activity. Galβ1–6Gal also had low or no inhibitory potency. Galβ1–3Gal, which occurs in pectic galactans as a minor disaccharide, inhibited galectin-3 and -9 with a potency similar to β-Lac and galectin-1 with a potency similar to Gal. Larger saccharides containing Galβ1–3Gal also had inhibitory potencies in the same range as Galβ1–3Gal. Other small saccharide components of pectic polysaccharides, such as GalA, Rha, or -4-linked internal Gal, are not known or expected to fit into the canonical galectin-binding site because of steric hindrance. Thus, the binding data of these small saccharides are consistent with the poor inhibitory potency of pectin polysaccharides of galectins.

##### Inhibition of Galectin-3-induced Hemagglutination

**FIGURE 3.**
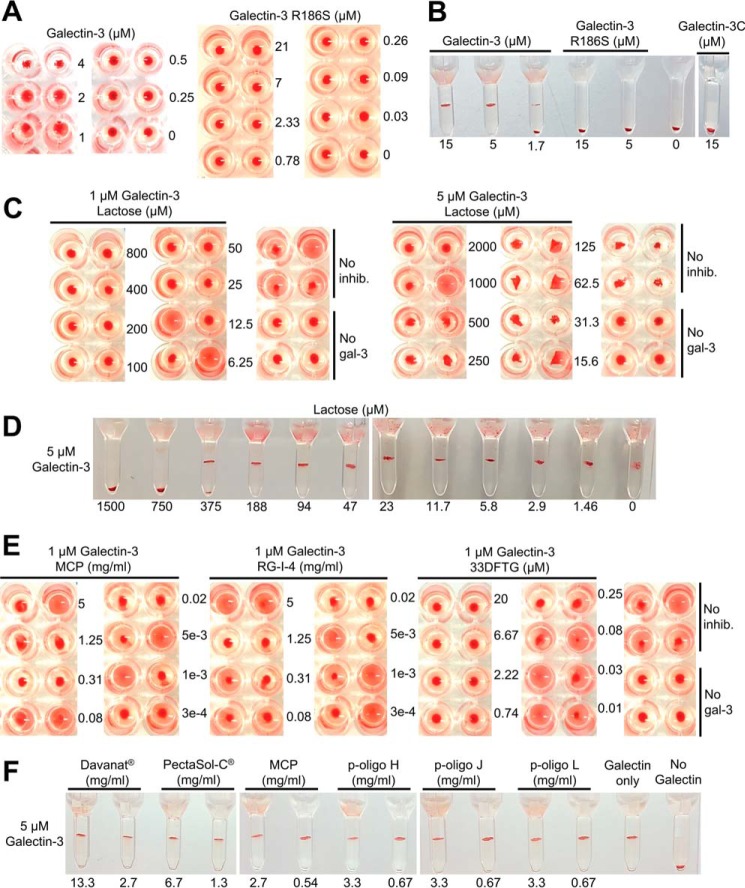
**No inhibition of galectin-3-induced hemagglutination, analyzed in V-shaped multiplates and gel cards, by different pectic samples and Davanat®.** Trypsin-treated and fixed hen erythrocytes (0.5–0.6% in NaCl solution) were used to study galectin-3-induced hemagglutination using either V-shaped plates or gel cards. The V-shaped plates containing the different erythrocytes/galectin/inhibitor mixtures were photographed 90 min after incubation (shaking for 90 min) of the plates. For the gel card experiments, the erythrocytes were first incubated (shaking for a short period) with galectin/inhibitor solutions in U-shaped plates before they were transferred and incubated in the gel card wells for 1 h, which were then centrifuged and photographed. *A,* efficacy of wild-type galectin-3 and the R186S mutant to induce hemagglutination in V-shaped plates. *B,* efficacy of wild-type galectin-3, the R186S mutant, and truncated galectin-3 lacking the N-terminal domain (galectin-3C) to induce hemagglutination, analyzed using gel cards. *C* and *D,* inhibition of galectin-3-induced hemagglutination with different concentrations of β-lactose analyzed using V-shaped plates (*C*) and gel cards (*D*). *E,* inhibition of galectin-3-induced hemagglutination in V-shaped plates (1 μm galectin-3) by modified citrus pectin (*MCP*), the rhamnogalacturonan sample (RG-I-4) (21), and a small molecule galectin-3 antagonist, 33DFTG (also known as TD139) (55). *F,* gel card analysis of galectin-3-induced hemagglutination inhibition by Davanat®, PectaSol-C®, MCP, galactan samples (p-oligo used in Ref. 21). All experiments in the V-shaped plates were done at least in duplicate, and most samples were additionally tested at least at two separate occasions. The samples tested in gel cards were tested in one well per gel card, and some of the samples were additionally tested at more than one occasion.

The poor inhibitory potency of some compounds in the FA assay was surprising, in light of published literature (17, 21, 38) that suggested a much higher potency to inhibit galectin-3-induced hemagglutination. Therefore, we analyzed galectin-3-induced hemagglutination and the effect of inhibitors in this assay in further detail. In the classical hemagglutination assay (Fig. 3, *A*, *C,* and *E*), erythrocytes are treated with agglutinin (or antibodies) in V-shaped microtiter wells and incubated for about an hour. With no agglutination (no galectin added), erythrocytes sink to the bottom of the well and are seen as a distinct button, whereas with agglutination, the aggregated erythrocytes get sticky and smear on the walls resulting in a mat covering the well walls or a less clear button (53).

This classical assay was originally developed for antibody-induced hemagglutination in blood group serology. As the readout depends on physicochemical interaction between the agglutinate and the well surface, it may be disturbed and difficult to read in the presence of complex large saccharides. Therefore, we applied gel cards as another hemagglutination assay, now used extensively in blood group serology (48) because of its much clearer readout (Fig. 3, *B*, *D* and *F*). In this assay, erythrocytes, agglutinin, and inhibitors are loaded on top of small gel-containing columns (with several held in a card). After centrifugation, large aggregates stay at the top, and free erythrocytes go to the bottom.

Galectin-3 was evident to induce hemagglutination both in the classical and gel card assay, where concentrations between 0.5 and 1 μm and 1.7–5 μm of galectin-3 were needed to induce clear hemagglutination in V-shaped plates and gel cards, respectively (Fig. 3, *A* and *B*). No agglutination in these assays was seen with galectin-3 mutant (R186S) (Fig. 3, *A* and *B*), with severely reduced affinity for LacNAc structures (lower part of Table 2) (43), or with truncated galectin-3 (galectin-3C) (Fig. 3*B*), lacking the N-terminal part of the protein and therefore having a reduced cross-linking ability (54).

Inhibition of galectin-3-induced hemagglutination was tested for different concentrations of β-Lac, in both V-shaped microtiter plates and gel cards (Fig. 3, *C* and *D*), to be used as reference values for the pectic samples and Davanat®. Results show (summarized in Table 3) that the MIC for β-Lac was dependent on the amount of galectin-3 used in the testing and in part by which hemagglutination assay was used (*i.e.* classical or gel card). The MIC for β-Lac was between 12.5 and 25, 200 and 500, 1000 and 2000, or >1500 μm for 1, 2, 5, and 10 μm galectin-3, respectively (Fig. 3*C* and Table 2). In the case of the gel card assay, the MIC for β-Lac was between 375 and 750 or 300 and 1500 μm for 5 and 10 μm galectin-3, respectively (Fig. 3*D* and Table 3). In the next step different pectins (PectaSol-C®, MCP, FPP, RG-I-4, and p-oligos) and Davanat® were tested for inhibition in either the classical or gel card assay or both of them for some samples; however, no inhibition could be observed at the concentrations tested (Fig. 3, *E* and *F,* and summarized in Table 3), except a partial inhibition observed for 5 mg/ml RG-I-4 when 5 μm galectin-3 was used. Additionally, a low molecular mass high affinity galectin-3 antagonist, 33DFTG (with *K_d_* value in the low nanomolar range, also known as TD139) (55), was tested in both hemagglutination assays and was shown to have MIC values between 0.25 and 0.74 μm for 1 μm galectin-3 and between 2.22 and 6.67 μm for 5 μm galectin-3 (classical assay) (Fig. 3*E* and Table 3), and a MIC between 2.22 and 6.67 μm for 10 μm galectin-3 (gel card assay) (Table 3).

**TABLE 3:** **Estimated MIC values for inhibition of galectin-3-induced hemagglutination in V-shaped plates and gel cards** As the MIC in this assay is somewhere between the concentration not able to inhibit the hemagglutination and the one able to do so, we define here the MIC values as concentration intervals (lowest concentration indicates no inhibition observed; highest concentration indicates no hemagglutination observed). NI indicates no inhibition at highest concentration tested. Blank field indicates not tested. NA means not applicable.

Samples	Highest [sample] tested	V-plate [galectin-3] (μm)				Highest [sample] tested	Gel card [galectin-3] (μm)	
		1	2	5	10		5	10
PectaSol-C® (mg/ml)	5 mg/ml	NI				6.7 mg/ml	NI	
FPP (mg/ml)	5 mg/ml	NI				NA		
MCP (mg/ml)	5 mg/ml	NI		NI		2.7 mg/ml	NI	
RG-I-4 (mg/ml)	5 mg/ml	NI		5*^a^*		NA		
p-oligo (mg/ml)	1 mg/ml	NI				NA		
p-oligo H (mg/ml)	1 mg/ml	NI				3.3 mg/ml	NI	
p-oligo J (mg/ml)	NA					3.3 mg/ml	NI	
p-oligo L (mg/ml)	NA					3.3 mg/ml	NI	
Davanat® (mg/ml)	5 mg/ml	NI				13.3 mg/ml	NI	
β-Lactose (μm)	2000 μm	12.5–25	250–500	1000–2000	1500*^a^*	1500 μm	375–750	300–1500
33DFTG (μm)	20 μm	0.25–0.74		2.22–6.67		20 μm		2.2–6.67

*^a^* Partial inhibition at the highest concentration was tested.

##### Analysis of the Relationship between MIC Value, Galectin-3 Concentration, and Inhibitor and Receptor Affinities

**FIGURE 4.**
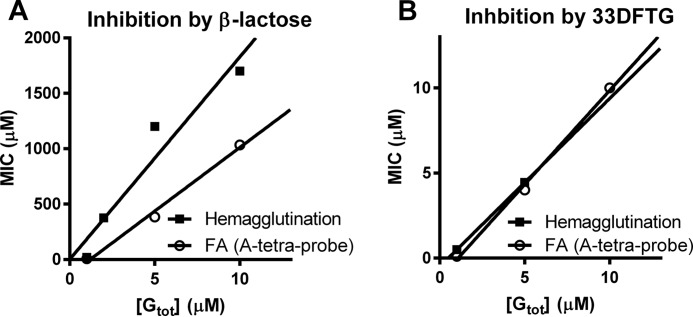
**Plotting of MIC *versus* the galectin-3 concentration used in the assay.** *A* shows inhibition by β-lactose with slopes of the lines about 100 or higher as described in text. *B* shows inhibition by 33DFTG with slope of the lines of about 1. For the FA assay, the saturation of target receptor (A-tetra-probe) was accurately determined from the measured anisotropy values, and the inhibitor concentration required to bring it under 50% (MIC) was found by interpolation between neighboring data. In the analogous way MIC of galectin-3-induced hemagglutination in V-shaped microtiter plates were estimated by interpolation between the lowest inhibitor concentration inhibiting hemagglutination, and the next lower concentration not inhibiting hemagglutination (Table 3). This is less accurate than for the FA assay, but it is still clear that with β-lactose as inhibitor the slope of the line is over 100 (*A*), whereas with 33DFTG it is closer to 1 (*B*).

The hemagglutination data clearly showed that with a higher concentration of galectin-3, more inhibitor was needed to prevent the hemagglutination. To understand this better, we derived an equation that relates a MIC value, defined as the inhibitor concentration bringing a fraction of putative receptors bound by galectin under 50%, with total added galectin concentration (as given under “Experimental Procedures” and legend to Fig. 4). With MIC value on the *y* axis and total added galectin on the *x* axis, this gives a straight line with a slope of 1 + (*K_d_*_I_/*K_d_*_R_), where the two *K_d_* constants are affinities of the galectin for inhibitor (I) and receptor (R).

To test this relationship experimentally, we first used the FA assay, where galectin affinity for inhibitor (*K_d_*_I_) and receptor concentration and affinity (*K_d_*_R_) represented by the fluorescent saccharide probe were known, and % receptor saturation could be easily measured as % bound probe from the anisotropy value. Note that the inhibitor concentration bringing total bound probe to a 50% “threshold” is a MIC value as defined for the FA assay, and it is not the same as IC_50_ value described above, at which binding with and without inhibitor was compared. The data agreed very well with the equation, as a weak inhibitor, like β-Lac, gave a very steep slope of about 100 (Fig. 4*A*), consistent with *K_d_*_I_ for β-Lac of about 90 μm and for the probe (*K_d_*_R_) of about 0.9 μm. With a much more potent inhibitor (33DFTG), with a *K_d_*_I_ ∼10 nm, the slope was close to 1 as expected (Fig. 4*B*). In both cases, the *x* axis intercept was about 1.2, in good agreement with its predicted value from the formula of [G_tot_] = 1/2[R_tot_] + *K_d_*_R_, when[I_tot_] = 0. After this validation, we plotted the MIC for β-Lac with galectin-3-induced hemagglutination. Here, the MIC was not as precisely defined as for the FA assay, as it is somewhere between the last dilution of β-Lac with inhibition and next without inhibition. Nevertheless, using estimates of these values, the data again were clearly consistent with a straight line. This now had slope of about 180. With known *K_d_*_I_ for β-Lac of about 90 μm, as mentioned above, this suggests that the average affinity for receptors on the erythrocyte surface (*K_d_*_R_) is <1 μm and/or the threshold receptor saturation may be less than 50%, as explained in the more generalized formula (Equation 8) under “Experimental Procedures.” The *x* axis intercept was significantly less than 1, consistent with *K_d_*_R_ and receptor concentration <1 μm. As expected, the slope for the 33DFTG inhibitor, in the hemagglutination experiments, was still around 1 (Fig. 4*B*) as *K_d_*_I_ is expected to be much smaller than *K_d_*_R_. Besides this, the equation, deduced under “Experimental Procedures,” also explains why it may be possible to inhibit a threshold-dependent (*e.g.* highly cooperative) effect, like hemagglutination, with very low concentrations of inhibitor, if the concentration of galectin added is just enough to raise receptor saturation over the threshold, and the concentration of inhibitor is just enough to bring it under the threshold again. In our case, for example, only 25 μm β-Lac was enough to inhibit hemagglutination induced by 1 μm total galectin, hence about 3 times lower than the *K_d_* value of β-Lac for galectin-3. With a *K_d_* value of 90 μm for β-Lac, this would give about 0.8 μm free galectin available for inducing hemagglutination (but failing to do so), showing how close to the threshold the assay was in this case.

Armed with the results and analysis described above, we asked whether it would be possible to reconcile the fact that some pectin-derived saccharides had no inhibitory potency in our FA assay when tested up a few milligrams/ml but were reported to inhibit hemagglutination at the microgram/ml level (17, 21). If the affinity of specific moieties in these polysaccharides for the galectin-3 canonical carbohydrate-binding site was low, for example *K_d_* ∼5 mm like Gal, which is about 50-fold worse than for β-Lac, then the slope of their line would be 50-fold steeper than for β-Lac. This would generate a slope of 7500, meaning that it would require 7500 times higher concentration of inhibitor to prevent hemagglutination if the galectin-3 concentration is increased with 1 μm in the assay. In Gao *et al.* (21), hemagglutination was done with slightly above 0.1 μm galectin-3, and the best pectic saccharide RG-1–4 had a MIC of 0.25 μg/ml and MCP had a MIC of 0.6 μg/ml. If their affinities were as exemplified above, with 1 μm more of galectin-3, their MICs would be about 2 and 5 mg/ml, respectively. Thus, it is possible to reconcile the data if the hemagglutination assay in Gao *et al.* (21) was done very close to the detectable hemagglutination threshold.

##### No Inhibition of Intracellular Galectin-3 Accumulation around Disrupted Lysosomes

**FIGURE 5.**
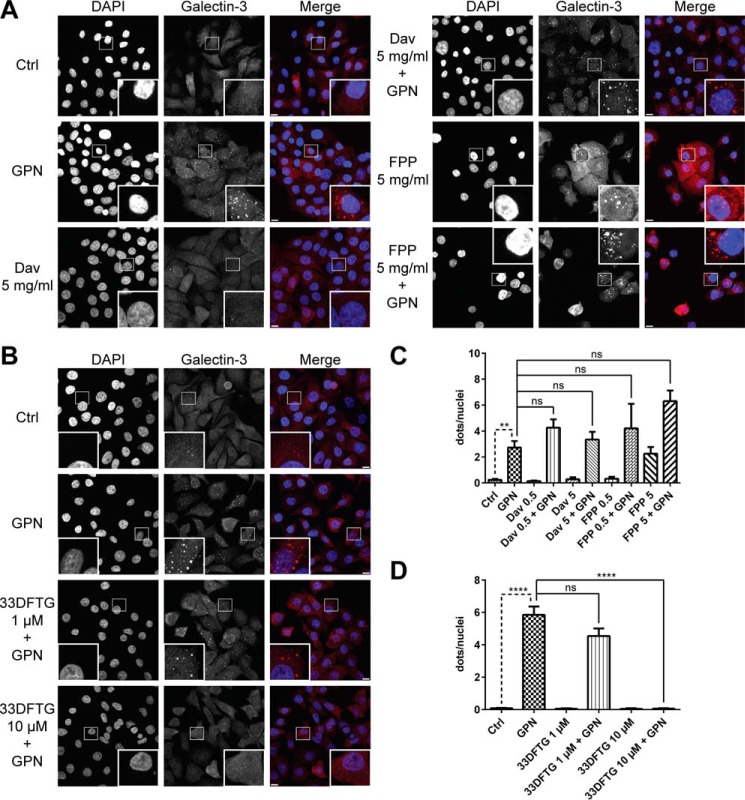
**No inhibition of galectin-3 accumulation around disrupted lysosomes by Davanat® or FPP in JIMT-1 cells.** *A* and *B,* representative images of JIMT-1 cells treated with 5 mg/ml Davanat®, FPP, or 33DFTG (as positive control) for 24 h before treatment with the lysosome-damaging agent GPN (0.3 mm for 12 min) known to induce accumulation of galectin-3 (58). *Scale bars* are equivalent to 10 μm. *C* and *D,* quantification of the number of galectin-3 dots/nuclei after treatment with the indicated concentrations of Davanat®, FPP, or 33DFTG for 24 h. The *bars* represent mean values obtained from 5 to 10 images from two independent cell cultures for each dataset ± S.E. A Student's *t* test (unpaired, two-tailed) was used to calculate statistical significance when the GPN-treated cells were compared with the control cells, whereas a Dunnett's test was used to evaluate statistically significant differences between the cells treated with GPN only and cells treated with Davanat®, FPP, or 33DFTG together with GPN. **, *p* < 0.01; ****, *p* < 0.0001, *ns,* not significant.

The anti-cancer effects of the different plant polysaccharides have sometimes been proposed to be mediated by extracellular galectin activities such as cell adhesion (1–6, 8, 9, 12–14, 17–19, 38) but also by intracellular effects such as the intracellular anti-apoptotic effect of galectin-3 (6, 10). Moreover, the polysaccharides are in many cases given orally in experimental treatments on animals (3, 5, 15, 22, 23, 56), suggesting that they are somehow taken up into cells. Therefore, we tested their effect on a defined intracellular galectin-3 activity known to require its carbohydrate binding activity, the accumulation of galectin around disrupted intracellular vesicles (57–59). To study this effect in a cell culture system, we induced lysosomal damage using GPN, which accumulates inside lysosomes and causes damage to the membrane via interaction with the lysosomal enzyme cathepsin C (60, 61) and induces galectin-3 accumulation (58). JIMT-1 breast cancer cells were pre-treated with the proposed inhibitor for 24 h, then treated with 0.3 mm GPN for 12 min to disrupt lysosomes, and then fixed and stained for galectin-3 (Fig. 5). If the putative inhibitors are taken up by the cells and have affinity for the galectin-3 CRD, they should be able to inhibit the galectin-3 accumulation caused by the GPN treatment, as seen for 33DFTG (Fig. 5, *B* and *D*) used as a positive control. With 0.5 or 5 mg/ml FPP or Davanat®, however, no inhibition of the GPN-induced galectin-3 accumulation could be seen for either compound (Fig. 5, *A* and *C*). FPP, however, was shown to induce galectin-3 accumulation by itself at the highest concentration (*p* value 0.0047, *t* test), which is not seen for Davanat®, indicating damage to a vesicular compartment in the cells treated with 5 mg/ml FPP (Fig. 5*A*).

##### Asialofetuin (ASF) Precipitation Assay

**FIGURE 6.**
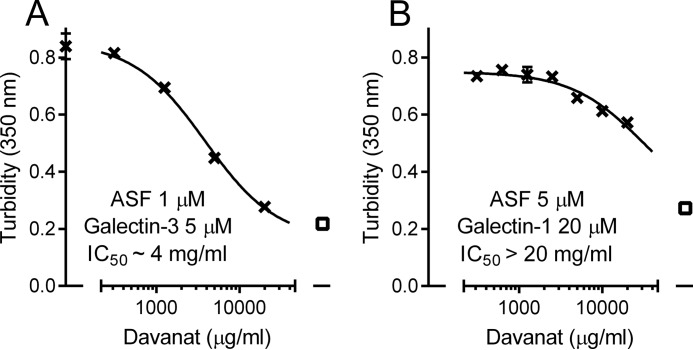
**Inhibition by Davanat® of galectin-1- or -3-induced aggregation with ASF.** Inhibition of galectin-3- (*A*) or galectin-1 (*B*)-induced aggregation of ASF by increasing concentrations of Davanat®, as measured by turbidity at 350 nm. The best fit calculated curve gives an IC_50_ value of ∼4 or 30 mg/ml, respectively. The *open square* indicates background absorbance at 350 nm in the absence of aggregates (*i.e.* buffer containing all components except ASF or galectin-3). All experiments were done in at least duplicate, and *bars* showing standard error are included but are not visible in most cases as they are smaller than the symbols.

Davanat® was additionally tested for its inhibitory potency of galectin-1- or -3-induced ASF precipitation (Fig. 6), as a model end point representing galectin/glycoprotein interaction. In this case, IC_50_ values were in the same range or slightly lower compared with the IC_50_ values for inhibition of the galectin probe binding. This suggests that the galectin/ASF interaction is fully explained by the availability of galectin with free carbohydrate-binding sites and indicates the absence of other mechanisms for this inhibition.

##### Davanat® Causes Oxidation-related Inactivation of Galectin-1

**FIGURE 7.**
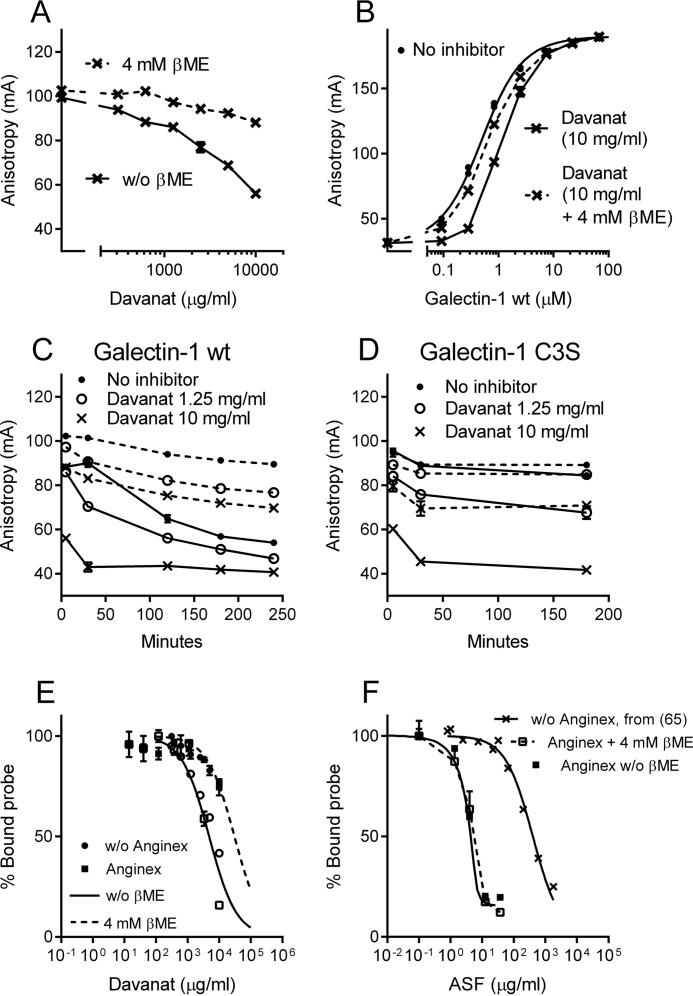
**Analysis of galectin-1 interaction with Davanat®.** *A,* inhibition of galectin-1 (0.5 μm) binding to tdga probe (0.1 μm) by increasing concentrations of Davanat®, measured by the FA assay. The experiment was done in the presence (*dashed line*) or absence (*unbroken line*) of βME as indicated. Measurements were done 5 min after mixing components. *Lines* connect data points. *B,* binding of increasing concentrations of galectin-1 wild type (*wt*) (*x* axis) to fluorescent tdga probe (0.1 μm) without (*filled circles*) or with Davanat® (10 mg/ml, ∼200 μm) as inhibitor (× *symbols*), in the absence (connected by *unbroken line*) or presence (*dashed line*) of βME. *C,* time course of galectin-1 WT/Davanat® interaction. The experiment was done as for *A*, but the results are shown for three Davanat® concentrations (0, 1.25, and 10 mg/ml) as read after different incubation times (5, 30, 120, 180, and 240 min) as indicated. The experiment was done in the presence (*dashed line*) or absence (*unbroken line*) of βME. *D,* time course of galectin-1 C3S/Davanat® interaction. The experiment was done as for *C*, except that galectin-1 WT was replaced by the relatively oxidation-resistant mutant C3S. *E,* inhibition of galectin-1 WT (0.5 μm) binding to tdga probe (0.1 μm) by increasing concentrations of Davanat® (*x* axis) in the absence (*circle symbols*) or presence (*square symbols*) of the peptide Anginex, with (*solid symbols*) or without (*open symbols*) βME. Best fit curves were modeled based on the data of *A*, showing that all data points in this experiment were also in close agreement with these curves, with (*dashed line*) or without (*unbroken line*) βME, respectively, and there was no effect of the presence of Anginex. *F,* inhibition of galectin-1 WT (0.5 μm) binding to tdga probe (0.1 μm) by increasing concentrations of ASF in the absence (*X symbols*, data from (65)) or presence (*square symbols*) of the peptide Anginex, with (*open symbols*) or without (*solid symbols*) βME. Best fit model curves are shown as *lines* for guidance. All experiments were done in at least duplicate, and *bars* showing standard error are included but are not visible in most cases as they are smaller than the symbols.

The interaction of Davanat® with galectin-1 was also examined in further detail (Fig. 7). As expected and well known, galectin-1 was prone to inactivation in the absence of a reducing agent, but unexpectedly, this inactivation was strongly accelerated by Davanat®. Thus, β-mercaptoethanol reversed most of the apparent inhibition of galectin-1 by Davanat® after 5 min (Fig. 7, *A* and *B*). Moreover, the activity of galectin-1, as measured by the FA value, gradually decreased over time after mixing sample components, the rate of the decrease correlated with the concentration of Davanat® present, and the presence of β-mercaptoethanol prevented most, but not all, of the galectin-1 inactivation (*dashed lines* in Fig. 7*C*). This is in striking contrast to the case with galectin-3 and galectin-9N, where no further change in the FA value read at 5 min was seen after the longer incubation times (up to 3 h), and the presence or absence of β-mercaptoethanol made no significant difference (Figs. 1*B* and 2*B*); this also provides evidence that the effect of β-mercaptoethanol is not due to a change of the Davanat®, but instead involves galectin-1, as expected. Galectin-1 contains six cysteines, and C3 has been found to be most prone to initiate oxidative inactivation, as the mutant C3S is relatively (but not completely) oxidation-resistant in some environments (51, 62). This mutant was relatively resistant to inactivation by the lower concentration of Davanat® for longer time periods but was equally sensitive to inactivation by Davanat® after 5 min of incubation (compare Fig. 7, *C* and *D*). Monomeric galectin-1 is most prone to oxidative inactivation, and dimeric galectin-1 is relatively resistant (51, 63). Consistent with this, the inactivation of galectin-1 by Davanat® was most pronounced at concentrations of galectin-1 below a few micromolars (Fig. 7*B*), where the monomer accounts for a significant part.

##### Investigation of Alternative Interactions between Pectins/Davanat® and Galectins

In a previous report (52), we showed evidence that a glycoprotein, like ASF, can induce galectin-3 self-association that engages the carbohydrate-binding site, which we named type-C to distinguish it from self-association mediated by the N-terminal domain of galectin-3 (36, 37). However, the same experiment as described above (Fig. 1, *C* and *D*) showed that Davanat® or PectaSol-C® did not induce type-C self-association of galectin-3. Had they done so, the binding curve in the presence of polysaccharide would have had a lower slope, which would not have reached saturation of the probe because more galectin would bind the ligand than expected from its number of binding sites (52). There was also no sign of precipitation (measured as absorbance at 350 nm) of Davanat® itself induced by either galectin-1 or -3, tested in a range of concentration combinations up to 10 mg/ml Davanat® and 100 μm galectin (data not shown).

We also tested the interaction of galectin-1 with Davanat® in the presence of the anti-angiogenic peptide Anginex (64), because we found it to have a very strong potentiating effect on galectin-1 interaction with fluorescein-tagged saccharide probes and with ASF and some other glycoproteins (65), possibly by interaction with the galectin at a site separate from the carbohydrate-binding site (66, 67). However, Anginex showed no influence on galectin-1 interaction with Davanat® (Fig. 7*E*), and Davanat® showed no influence on the Anginex effect on galectin-1/probe interaction. In contrast, as shown here for comparison, Anginex potentiated the inhibition of galectin-1 by ASF by a factor of about 500, and this occurred equally with or without β-mercaptoethanol (Fig. 7*F*).

In a last assay we wanted to see whether Davanat®, PectaSol-C®, or FPP could inhibit the interaction of the NDP52-derived peptide and galectin-8, as clearly demonstrated by Kim *et al.* (46), using an FA assay and reproduced with similar results in our study (data not shown). NDP52 interacts with galectin-8 at a site located opposite to the C-terminal carbohydrate-binding site, similar to the interaction reported for Davanat® and galectin-1 and -3 (34, 40). If they bound such a site on galectin-8C, they might be able to inhibit the interaction with the NDP52 peptide. No inhibition could, however, be seen of the NDP52/galectin-8 interaction by either Davanat®, PectaSol-C®, or FPP (data not shown) when used at concentrations ranging from 0.03 to 1.67 mg/ml.

## Discussion

**FIGURE 8.**
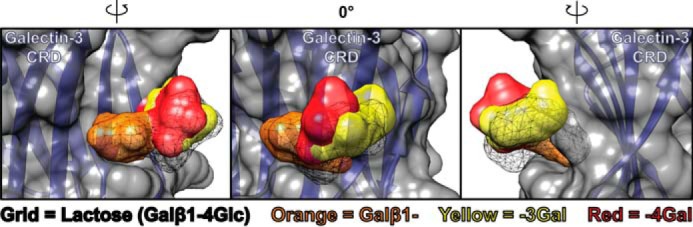
**Three-dimensional structure of the CRD of galectin-3 in complex with β-lactose (Lac) and the theoretical positioning of Galβ1–3Gal and Galβ1–4Gal.** The non-reducing end galactose moiety (shown in *orange*) of the two galactobioses was aligned with the galactose residue of the β-Lac molecule (shown as grid). This results in different positions of the other saccharide moiety, reducing end Gal, as shown in *yellow* and *red*, respectively, for each galactobiose. It is clear that Galβ1–4Gal is less similar to β-Lac in this part (*red*), and it interacts less well with the galectin CRD. The coordinates of galectin-3 in complex with β-Lac were from Protein Data Bank code 3ZSJ, and d-Gal-β-(1,3)-d-Gal and d-Gal-β-(1,4)-d-Gal saccharides were obtained from the SWEET II saccharide generator (76). The modeling and alignment were done using PyMOL Molecular Graphics System version 1.7.0.3 (Schrödinger LLC, New York), and the final pictures of the galectin-3·saccharide complex were generated using UCSF Chimera version 1.9 (University of California at San Francisco) (77).

There is a large body of evidence that the defining canonical galactose-binding site of galectins plays a central role in their function (36). Inhibition of this site, in particular in galectin-3, has been proposed or claimed as the mechanism behind the interesting anti-cancer and anti-inflammation effects of certain plant-derived polysaccharides (1–6, 9–16, 18, 19). Now, however, we show that several commonly used “galectin-binding glycans” are very poor inhibitors, or not inhibitors at all, of the canonical binding sites of purified galectins in solution. This poor inhibitory potency is consistent with the known biochemical analysis of the polysaccharides. RG-I-4, FPP, PectaSol-C®, and Davanat® are polydisperse with molecular mass ranges of about 60, 20–60, 10–20, and 40–60 kDa, respectively, which translates on average to 300, 200, 100, and 300 monosaccharides per molecule, respectively. Most of these monosaccharide residues would not bind the defining galectin-binding site (named site C in Salomonsson *et al.* (43)) because either they do not have the galacto configuration or, if they do, substitutions at positions 4 and/or 6 would prevent binding. Some Gal residues are available for binding, and they are in most cases predicted to bind galectin with about the same or poorer affinity as free Gal (*K_d_* ∼10 mm). This is the case for Galβ1–4Gal, which is the most common galactose-containing disaccharide moiety in pectin-derived glycans, and Galα1–6Man, which is most common in galactomannans (as tested here, see Table 2). Stronger galectin binding can be seen if the Gal is linked to the next residue in such a way that the latter occupies the adjacent binding site (site B or D in Salomonsson *et al.* (43)) in a stereochemically favorable way. This is the case with, for example, the glucose (Glc) residue, -4Glc in Lac, and the second Gal in Galβ1–3Gal, which boosts affinity by about 30–100-fold (see Table 2). However, such residues (Galβ1–3Gal) are rare in modified citrus pectins (<1%) and not found in Davanat® (8, 34, 39). Structural modeling of Galβ1–4Gal and Galβ1–3Gal into the canonical binding site (sites C and D) of galectin-3 is consistent with the different affinities (Fig. 8), where the second Gal of Galβ1–4Gal (*red* in Fig. 8) does not interact much with the protein and may even have a small steric conflict (most clearly seen in *left panel* of Fig. 8). In contrast, the second Gal of Galβ1–3Gal (*yellow* in Fig. 8) can be accommodated well in site D, as previously shown for GalNAc in Galβ1–3GalNAc (68). Galβ1–3Gal could also fit in sites B and C. Thus, the biochemical data suggest that canonical galectin inhibition by pectin and galactomannan polysaccharides is mainly due to weak interaction with multiple Gal residues, as is also consistent with our titration data for galectin-3 (Fig. 1, *C–E*).

This agrees with the conclusion from detailed studies of pectin fragments by Gao *et al.* (21). The activity of the most potent large fragment, RG-I-4, was proposed to be due to multiple Gal-containing side chains, even if each side chain by itself had much lower activity. The FA assay here measures the ability of inhibitors to compete with the carbohydrate-binding site of galectin molecules in solution, “one at a time,” because multivalency effects may not be seen. In other direct binding assays, where much higher galectin binding activities were suggested, either the galectin or the plant polysaccharide was coated on a surface (2, 17, 21, 29, 38), and although binding could be demonstrated, the affinity and the role of the canonical carbohydrate-binding site remain unclear. Other interactions may also contribute, such as charge, because citrus pectin-derived saccharides have multiple negative charges due to GalA, and some galectins, including galectin-3, have high isoelectric points making them strongly positive at neutral pH. Thus, for example, interaction in surface plasmon resonance was seen with the galactose-depleted backbone of RG-I-4 (21).

Regarding the inhibition of galectin-3-induced hemagglutination, there is a clear discrepancy between the present results, with poor inhibition by the pectin fragments, and the results by Gao *et al.* (21), with strong inhibition by many fragments. One explanation may be that here higher galectin concentrations were used (1–10 μm compared with about 0.1 μm) and higher temperature (about 23 °C compared with 4–17 °C). The more sensitive “tuning” of the assay delimitation between agglutination and non-agglutination may have made it more sensitive to lower concentrations of pectin fragments even if these have low affinity for the galectin, as we show in our theoretical consideration (Fig. 4). In this study, the hemagglutination was clearly shown to depend on the carbohydrate binding activity of galectin-3 as the mutant R186S had no effect. Thus, it is also clear that even a large excess of pectin fragments is not able to block the carbohydrate binding or other hemagglutination inducing activity of 1–5 μm galectin-3 in this assay. Had they had high affinity for galectin-3, the slope of the line seen in Fig. 4 would have been lower, and one would have expected inhibitory activity also at some of the higher concentrations tested here.

The poor galectin inhibitory potency of the polysaccharides analyzed here is not, as mentioned above, consistent with the biological effects proposed to be due to galectin inhibition. At the concentrations typically used to induce cancer- or inflammation-related effects in cell culture or *in vivo* (<5 mg/ml), the polysaccharides analyzed here show no inhibition of the carbohydrate binding activity of purified galectins in solution. There are several possible reasons for this discrepancy.

The higher galectin inhibitory potencies of the plant polysaccharides reported by others are mainly based on indirect assays that include incubation with cells over extended times. Neither of these show that the measured effect is due to binding of the plant polysaccharide to galectin-3, because there are many other effects that could have occurred during the long incubation and in turn affected the localization of the galectin. The externalization and cell surface exposure of galectin-3 are regulated by lipopolysaccharides (69, 70), for example, and external galectin-3 is taken up into cells within minutes and is involved in a rapid, probably regulated, recycling between the cell surface and intracellular vesicles (71, 72). An interesting and theoretically possible effect on galectins of the plant polysaccharides would be if somehow their weak binding trapped the galectin extracellularly, thereby depleting it from the cell or tissue over time. This could not work simply based on their weak affinity for the galectin and law of mass action; additional mechanisms such as energy-requiring or rate-limiting steps would have to be included, and this is an interesting possibility to explore.

Plant polysaccharides such as modified citrus pectin and galactomannan are not expected to be taken up into cells across membranes, but they may very well be endocytosed, as for example is well known for dextran (73), which often is used as a tracer for macro- and micro-pinocytosis and delivered into lysosomes. Here, we preloaded cells with either Davanat® or FPP for 24 h, after which they would be expected to have accumulated in lysosomes. Then we disrupted lysosomes with GPN and looked for the known accumulation of galectin-3 (57–59). The pre-incubation with Davanat® or FPP had no effect on this. However, FPP by itself induced some galectin-3 accumulation around intracellular vesicles. Thus, one may speculate that one indirect effect of the plant polysaccharides on galectins may be to affect intracellular galectin distribution by disrupting intracellular vesicles. However, this remains to be proven.

The possible interaction of a plant polysaccharide with another binding site on a galectin has been most clearly demonstrated by NMR for the binding of Davanat® to high concentrations galectin-1 and -3 (34, 39, 40). Davanat® induced band broadening in the NMR of the galectins suggesting binding at another site, outside the canonical β-galactoside-binding site, possibly with higher apparent affinity of *K_d_* ∼10 μm for galectin-1. Conversely, it was concluded that Davanat® at 0.35 and 0.7 mg/ml did not compete with canonical binding of β-Lac to 70 μm galectin-1 as analyzed by chemical shift perturbation (34), which is consistent with the high IC_50_ value for Davanat® measured here (Table 1). In another recent report by Miller *et al.* (40), Davanat® (named GM 1.7) was proposed to have about six sites for non-canonical binding to galectin-3 with an average affinity of about 17 μm. Although our FA assay mainly measures interaction with the canonical carbohydrate-binding site, the interaction of Davanat® or PectaSol-C® with a non-canonical site of *e.g.* galectin-3 tested would theoretically have been seen as a rise in anisotropy in the FA assay, due to the increased size of the protein·probe·polysaccharide complex. This was however not seen in this study, as exemplified in Fig. 1*A* for galectin-3, where the curves for both Davanat® and PectaSol-C® follow a straight line up until the beginning drop in anisotropy seen around 200 and 3000 μg/ml, respectively (due to decreased binding of the fluorescent probe).

To assess the possible contribution of such non-canonical sites in galectin-mediated glycoprotein cross-linking, we analyzed aggregation of ASF induced by galectin-1 and galectin-3. The concentration of Davanat® that inhibited the ASF aggregation by 50% (4 mg/ml for galectin-3 and >20 mg/ml for galectin-1, Fig. 6) was similar to the concentrations that inhibited in the binding of fluorescent probe to the canonical carbohydrate-binding site of either galectin (Table 1), and it was much higher than what gave 50% interaction (∼0.5 mg/ml) at the alternative site in galectin-1 or -3 suggested by NMR (34, 40). Therefore, this alternative binding site is not likely to be rate-limiting for the cross-linking of ASF by galectin-1 and -3. Instead ASF aggregation by galectins-1 and -3 depends mainly on the canonical β-galactoside-binding site (51, 52).

We also found that Davanat® induced inactivation of galectin-1, most likely oxidative, as it was reversed by β-mercaptoethanol. As this effect was most pronounced at low galectin-1 concentrations (low micromolar range) where the galectin-1 monomer is a significant component, it may be significant in a physiological situation. It may not have been seen in the NMR studies mentioned above, where much higher concentration was used, where galectin-1 is almost exclusively dimeric. Thus enhanced oxidative inactivation of galectin-1 or other proteins is another possible mechanism of action of plant polysaccharides.

In conclusion, our data do not support the widely reported proposal that the beneficial effects of pectins and galactomannans have their effect mainly as inhibitors of the canonical carbohydrate-binding site of galectins. It is therefore not experimentally justified to regard them as specific galectin inhibitors. Instead, additional or alternative mechanisms must be involved; these may include interaction with other sites on galectins, as suggested for Davanat® (although no evidence for such interactions was found in this study), or interaction with other types of molecules altogether (as demonstrated by Leclere *et al.* (74, 75)). Given their interesting biological effects, elucidating the mechanism of action of such plant polysaccharides is of great interest, and now perhaps the focus should be expanded beyond galectins.

## Author Contributions

Fluorescence anisotropy and turbidimetry studies were performed by B. K.-K., J. S., and A. L. Galectin-3-induced hemagglutination studies were done by J. S. and B. K.-K. J. S. and S. O. performed the *in vitro* vesicle damage studies. M. A., R. A. F., and A. A. K. produced some of the pectin and galactomannan samples analyzed in the study. U. J. N. and co-workers synthesized the fluorescent saccharide probes used in the FA assay. H. L. designed and supervised the project. The writing of the manuscript was mainly done by J. S., A. L., and H. L. with contributions and constructive feedback from all authors.

## Footnotes

5 M. Aguilar-Moncayo and R. A. Field, personal communication.

## Footnotes

4 The abbreviations used are:

MCP: modified citrus pectin
FPP: fractionated pectin powder
GalA: galacturonic acid
RG: rhamnogalacturonan
Rha: rhamnose
CRD: carbohydrate recognition domain
FA: fluorescence anisotropy
Lac: lactose
LacNAc: *N*-acetyl-lactosamine
33DFTG: 3,3′-dideoxy-3,3′-bis-[4-(3-fluorophenyl)-1*H*-1,2,3-triazol-1-yl]-1,1′-sulfanediyl-di-β-d-galactopyranoside
GPN: glycyl-l-phenylalanine 2-naphthylamide
FBS: fetal bovine serum
MIC: minimum inhibitory concentration
ASF: asialofetuin
A-tetra: GalNAcα1–3(Fucα1–2)Galβ1–4Glc
tdga: thiodigalactoside-amide used in one of the probes
βME: β-mercaptoethanol.
